# National expert consensus on home-administered oncologic therapies in Spain

**DOI:** 10.3389/fonc.2024.1335344

**Published:** 2024-02-16

**Authors:** Eulalia Villegas, María Arruñada, Miguel Ángel Casado, Sonia González, María Estela Moreno-Martínez, María Ángeles Peñuelas, Ana Maria Torres, Yanik Sierra, Miguel Angel Seguí

**Affiliations:** ^1^ Dos de Maig Hospital, Barcelona, Spain; ^2^ Pharmacoeconomics & Outcomes Research Iberia (PORIB), Madrid, Spain; ^3^ Innovation in Clinical Pharmacy Research Group (i-FARMA-Vigo), Vigo, Spain; ^4^ Galicia Sur Health Research Institute (IIS Galicia Sur), Vigo, Spain; ^5^ University Hospital Complex of Vigo (SERGAS-UVIGO), Vigo, Spain; ^6^ Pharmacy Service, de la Sant a Creu i Sant Pau Hospital, Barcelona, Spain; ^7^ Vall d'Hebron University Hospital, Barcelona, Spain; ^8^ Eisai Farmaceutica SA, Madrid, Spain; ^9^ Parc Taulí Foundation, Barcelona, Spain; ^10^ Autonomous University of Barcelona, Barcelona, Spain

**Keywords:** oncology, home administration, expert recommendations, quality of life, oncology therapies

## Abstract

The diagnosis and treatment of cancer impose a significant emotional and psychological burden on patients, families, and caregivers. Patients undergo several interventions in a hospital setting, and the increasing number of patients requiring extended care and follow-up is driving the demand for additional clinical resources to address their needs. Hospital at Home (HaH) teams have introduced home-administered oncologic therapies that represent a new model of patient-centered cancer care. This approach can be integrated with traditional models and offers benefits to both patients and healthcare professionals (HCPs). Home-administered treatment programs have been successfully piloted globally, demonstrated as a preferred option for most patients and a safe alternative that could reduce costs and hospital burden. The document aims to establish the minimum recommendations for the home administration of oncologic therapies (ODAH) based on a national expert agreement. The expert panel comprised seven leading members from diverse Spanish societies and three working areas: clinical and healthcare issues, logistical and administrative issues, and economic, social, and legal issues. The recommendations outlined in this article were obtained after a comprehensive literature review and thorough discussions. This document may serve as a basis for the future development of home-administered oncologic therapy programs in Spain.

## 1 Introduction

Cancer remains a major global health problem despite scientific efforts in the development of new therapies (1, 2). According to GLOBOCAN data, 19.3 million new cases and 10 million cancer deaths were reported in 2020 worldwide, and a 47% increase in new cases will occur in 2040 if the current rates remain constant (3). In Spain, 277,394 new cases were diagnosed in 2020, currently being the second cause of death and accounting for 22.2% of total deceases (109,706 cancer deaths). According to Spanish Network of Cancer Registries (REDECAN) data, 280,100 newly diagnosed patients are expected by the end of 2022 (4).

Patients with cancer undergo multiple interventions, typically performed within a hospital setting that provides the requisite logistical resources. The reliance on the hospital environment has been amplified by the necessity for a multidisciplinary team of healthcare professionals (HCPs) and the development and deployment of new technologies for diagnosis and treatment. Nevertheless, the COVID-19 pandemic has had adverse effects in various routine clinical procedures due to hospital saturation, insufficient resources, and recommended restrictions aimed at reducing the risk of infection (5–7). Consequently, the regular activity of the oncology units was affected (6, 7). A meta-analysis reported a reduction in general clinical activity in 58% of the centers worldwide during the first wave (6). Frequently, treatment delay or cancellation, change in treatment plans, and delay in outpatient visits (in 58%, 65%, and 75% of centers, respectively) were registered, and many centers (72%) implemented virtual visits (6).

Hospital at Home Service (HaH) is hospital-level substitutive care delivered at home for acute patients who required hospital admission. HaH has been associated with several advantages, including patient safety, reduction of nosocomial complications, similar or even better health outcomes compared to conventional hospitalization, high satisfaction levels from both patients and caregivers, and cost savings (8, 9).

HaH teams are trained to perform complex interventions at home, which would reduce the hospital burden preventing the negative consequences of this overload on cancer patients (10–13). Moreover, treating cancer patients at home can help to control high-risk situations such as the exposure to epidemics of multidrug-resistant pathogens or community viral infections with high morbidity and mortality (14).

Despite the growing interest, the available literature exploring the possibilities and benefits of oncologic drug administration at home (ODAH) and supportive care treatments in oncology patients is still limited and controversial, especially in Spain. The aim of the present document is to establish the minimum recommendations for an ODAH based on a national expert consensus. It should be noted that in the present document this setting refers to all healthcare-related procedures coordinated by a multidisciplinary group of HCPs that attend to the diagnostic and therapeutic needs of selected oncology patients at their homes. This home-based care is a complementary element of the protocol designed for those patients and, thus, it must be integrated as part of it. Due to their singularities, pediatric oncologic patients and those with haemato-oncologic diseases are not considered in the elaboration of this recommendations.

## 2 Materials and methods

### 2.1 Expert selection and panel composition

The panel of experts was multidisciplinary and included seven professionals from Madrid, Barcelona and Vigo involved in cancer treatment and who belong to diverse scientific societies: SEFH (Spanish Society of Hospital Pharmacy), SEHAD (Spanish Society of Home Hospitalization), SEEO (Spanish Oncology Nursing Society), and SEOM (Spanish Society of Medical Oncology). The panel included experts in the field of oncology, nursing, HaH, hospital pharmacy, and health economics. Three working areas were defined: 1) clinical and healthcare issues, 2) logistical and administrative issues, and 3) economic, social, and legal issues. Experts were assigned to a working group according to their knowledge and expertise (**Supplementary Table 1**).

### 2.2 Literature screening and questions formulation

A series of relevant questions for each section were prepared (see **Supplementary Table 2**). The experts reviewed the literature and provided individual responses, but a systematic review was not conducted. The working group then deliberated on the responses and arrived at a consensus on the recommendations. The decisions were primarily guided by expert opinions and were reinforced by the existing literature. In instances where evidence was lacking, only expert opinions were taken into account.

### 2.3 Consensus meeting and agreement

The consensus meeting took place on November 16th, 2022. The recommendations of each working group were presented to all experts and discussed in the meeting to reach a final agreement. The present recommendations are presented in question-answer format and have been endorsed by all participants. The role and functions of the main stakeholders involved in home administration of oncologic therapies were defined and summarized in **Supplementary Table 3**.

## 3 Results

### 3.1 Section 1: clinical and healthcare issues

When considering the feasibility of a home-based oncology treatment program, it is important to take into account various factors related to the oncology drug, therapy, patient characteristics, and healthcare environment.

#### 3.1.1 Item A. Related to drug/therapy

**Question 1. Which drugs are potentially suitable for home administration?**

When planning a home-based oncology treatment program, it is important to consider the pharmaceutical and clinical characteristics of the administered treatment, including its posology, stability, route and duration of administration, and safety profile. Most articles describing ODAH focus on parenterally administered treatments, with oral medications not typically considered as home-administered chemotherapy (12, 15). Oncologic therapies that have a non-complex administration protocol and a known and manageable safety profile are good candidates for home-base administration (15). The duration of chemotherapy treatment typically varies from two to eight months, and shorter administration times are generally preferable to longer ones (16). Prolonged home-administered therapy, especially if it requires the presence of a nurse throughout the entire procedure, can result in a waste of resources for hospitals and increase costs (15, 17, 18). According to published initiatives, the infusion duration of home-administered drugs usually took less than four hours (15). Moreover, guaranteeing drug stability for its administration at home is essential to ensure the best treatment for the patient (19).

An indicative list of oncologic drugs that can potentially be administered at home is presented in **Table 1**, although there is significant variability in the types of antineoplastic drugs used in home-based therapy programs (12, 15, 20–22).

**Table 1:** Potential oncologic treatments for home administration.

Drug	Posology	Ad. route	Premedication
5-Fluorouracil	15 mg/kg or 600 mg/m^2^ once a week (initial treatment) *	IV	None
Eribulin	1.23 mg/m^2^ (days 1 and 8 every 21-day cycle)	IV	None
Methotrexate	single doses ranged 20-40 mg (10-20 ml)/m² BSA*	IM/SC	None
Nivolumab	240 mg/2 weeks (~30 min) * 480 mg/4 weeks (~60 min) *	IV (not push or bolus)	None
Pembrolizumab	200 mg/3 weeks 400 mg/6 weeks	IV	Antiemetics
Pemetrexed	500 mg/m^2^ BSA*	IV	Corticosteroids
Pertuzumab	Initial dose of 840 mg (~60 min) Maintenance dose 420 mg/3 weeks (~30-60 min)	IV	None
Trabectidin	1,5 mg/m2 BSA (over 24 h, 3-week interval between cycles) *	IV	None
Trastuzumab	Dose and regimen depend on indication (weekly or 3-weekly schedules)	IV (not bolus)/SC	None

BSA, body surface area; EPOCH, etoposide, vincristine, doxorubicin, cyclophosphamide, and prednisone; IM, intramuscular; IV, intravenous; SC, subcutaneous.

*Dose and regimen may depend on indication, patient’s condition, or previous/concomitant treatment.

#### 3.1.2 Item B. Related to patients

**Question 2. What profile of patients would benefit from oncology treatment administered at home?**

Eligibility criteria for ODAH is crucial to maintain the safety and should be based on patient and caregiver readiness, diagnosis, characteristics and co-morbidities, treatment regimen, and hospital proximity and home environment (**Table 2**) (15, 16). Inclusion criteria should be related to the clinical and physical state, cancer type and grade, and type of treatment. External factors, such as the distance to the hospital, presence of a caregiver/relative and housing conditions should also be assessed by a multidisciplinary team of HCPs (16).

**Table 2:** Recommended inclusion and exclusion criteria to select the most adequate patients for home-administered chemotherapy.

Inclusion criteria	Exclusion criteria
Diagnosis of oncological disease requiring systemic treatment.	Lack of availability or capacity of the HaH to undertake the procedure.
Patient of legal age and with capacity to make autonomous decisions.	Severe delayed toxicity associated with treatment or prior requirement for medical attention.
Explicit acceptance of the on-home administration care resource by the patient or his/her legal guardian.	Clinical instability that limits the procedure.
Geographical area within the responsibility of HaH.	Pharmacological treatments with risk of drug-drug interaction after assessing that is a threat to the patient.
Previous administration of medication in the hospital centre without serious adverse reactions.	Inclusion in a clinical trial.
1-3 treatment cycles without incident.*	Patients with risky behavioural alterations.
General clinical stability with no evidence of acute intercurrent conditions.	Unhealthy or unsanitary conditions at home.
Mobility problems or severe functional disability that makes movement difficult.	
Difficulty of the patient to reconcile work or family life in attending the Day Hospital.	
Comorbidities that make access to the Day Hospital difficult or inadvisable.	
Accompaniment of the patient during the intervention.	
Existence of a trained primary caregiver and its compliance in case of disabilities.	
Availability for telephone communication or teleassistance.	

*The minimum number of cycles it will depend on the selected drug and individual patient evaluation.

#### 3.1.3 Item C. Related to healthcare settings

**Question 3. How would ODAH compromise the efficacy, safety, and quality of life of systemic treatments?**

Research has shown that home-administered oncology treatment can be just as effective and safe as hospital-administered treatment and may lead to improvements in quality of life and patient satisfaction (12, 15, 16). Recently, a systematic review found that ODAH presented no statistically significant differences in quality of life compared to hospital administered setting (12, 23). Safety data showed that toxicities were expected regardless of the location of administration. In Spain, a randomized controlled trial comparing home-based chemotherapy to hospital treatment revealed higher satisfaction with home-based administration in terms of the perception of nursing availability and communication (24). Another systematic review, exploring the advantages of home chemotherapy pointed that the results obtained in several trials sustained that treatment administration at home was safe and feasible, and preferred by patients and caregivers (25).

However, some clinicians may still have concerns about patient safety (26), but the appropriate preparation and education can help ensure the safety of the procedure (16). Patients and caregivers should receive educational and supporting material that explains the program implications in simple and easy-to-understand vocabulary. Periodic satisfaction surveys can also help to monitor the patient experience and identify areas for improvement. Also, further research is needed to compare the efficacy, safety, and quality of home-administered therapy to hospital treatment.

**Question 4. What kind of education, accreditation or training should receive the HCPs involved?**

Oncology, HaH and Pharmacy Services must be coordinated for drug prescription, validation, evaluation of the clinical state before drug administration, detection of adverse events, and patient follow-up. Therefore, an interdisciplinary group of HCPs with defined responsibilities is required along with specialized education and training (15, 27).

Furthermore, antineoplastic drug administration is a complex procedure that must be performed by a qualified professional to avoid undesirable incidents (28–31). Specialized nurses are responsible for the chemotherapy and biotherapy administration, and they have specific education and training that ensure the safe care of the patient (28, 32).

Implementing this strategy will typically necessitate the establishment of specialized teams dedicated to administering antineoplastic treatments in patients’ homes. While it is feasible to utilize home hospitalization resources or hospital nursing to a certain extent, this approach generally entails a greater demand for human and material resources.

**Question 5. What kind of controls should be performed?**

Experts suggest and agree that certain minimum requirements must be met by the hospital when considering a ODAH program: 1) to have a Medical Oncology Service; 2) to have a territorial/regional HaH Service; 3) to have a Pharmacy Service that ensures the highest quality of care for patients; 4) to be provided with minimum healthcare resources in the units and available involved Services; and 5) to perform a demographic and geographic evaluation of the healthcare area.

An initial pilot program should be set up to evaluate the results and determine the compliance of requirements. Standardized approaches and procedures, along with interdisciplinary professional review, can help avoid medication mistakes (28–30). An indicative checklist is provided in **Table 3** and a standardized nurse visit is shown in **Supplementary Figure 1**.

**Table 3:** Checklist of the optimal steps to perform the ODAH.

Time point	To check/perform:
Before administration	- Clinical state (diagnosis and treatment scheme) - Prescribed medication (antineoplastics, concomitant, premedication). * - Patient medical record (allergies, comorbidities, venous accesses, etc). - Need of previous medical controls. - Administration system required. - Patient’s identity. - Administration scheme.
During administration	- Assure patient’s identity and administration scheme. - Periodically check the adverse events and administration route state. - Clinical and hemodynamic stability. - Ask about previous tolerance to treatment. - Give recommendations about toxicity.
After administration	- Venous/cutaneous state (e.g., ensure for no extravasation). - Healing/catheter registration. - Ensure the patient understand the recommendations for toxicities detection. - Register necessary information in the medical record. - Adequate medical waste disposal.

*Verification by at least two health-care professionals.

**Question 6. What would be the minimum instruments, apparatus or equipment required?**

**Table 4** includes a list of materials to be considered for an ODAH program. The minimum required material will depend on the therapy and clinical state of the patient (18).

**Table 4:** Basic and additional materials needed for ODAH procedure.

Category	Materials
Basic	- Reference manual for the ODAH. - Extravasation kit. - Spill kit. - Emergency kit. - Venepuncture material. - Medical prescription. - Computer or similar for informatic access. - Material for waste management. - Sphygmomanometer, pulse oximeter, and stethoscope. - Temperature-controlled box for dug transport. - Basic life support.
For venous cannulation	- Endovenous catheter. - 10cc saline solution injections (pre-filled or not). - Sterile gauzes and dressings, antiseptic, absorbent towel. - Container for sharp objects. - Port-a-catch needle. - Catheter Abbocath No. 22. - Adhesive skin sutures. - Sodium heparin for catheter flushing. - Healing material for peripheral venous catheter.
Visiting HCPs equipment	- Protection material (gloves, mask, disposable gown, glasses). - Container group IV.
Vehicle equipment	- Hermetic refrigerator for transport of cytostatic drugs. - Container for cytostatic drugs. - Emergency kit. - Infusion pumps. - Container group IV and for sharp objects.
Diagnosis material (HBH Service)	- Electrocardiogram. - Pulse oximeter. - Automated external defibrillator (AED). - Necessary equipment for sample collection and analysis.
Additional therapeutic material (HBH Service)	- Oxygen therapy. - Aerosol therapy. - Elastomeric and perfusion pumps. - Transfusions of blood components. - Material for ostomy and tracheostomy management. - Mechanical ventilation instruments (BiPAP, CPAP). - Digit puncture for measurement of INR. - Material for enteral or invasive nutrition. - Surgical drainages. - Bladder catheterization. - Replacements of probes and cannulas. - Negative pressure therapy treatments. - Advanced life support.

AED, Automated external defibrillator; BiPAP, Bi-level Positive Airway Pressure; CPAP, Continuous Positive Airway Pressure; HBH, home-based hospitalization; HCPs, healthcare professionals; INR, international normalized ratio.

**Question 7. Which aspects should be considered for drug preparation?**

To ensure the best treatment for the patient, it is crucial to maintain drug stability during home delivery. This requires a review of all factors involved in drug preparation and transport, and the implementation of protocols and controls to assess drug stability for optimal implementation of a home-based therapy program (19).

#### 3.1.4 Recommendations of the experts panel for the section 1

**Table 5** contains the agreed recommendations made by the panel of experts regarding clinical and healthcare issues section.

**Table 5:** Expert recommendations for clinical and healthcare issues.

Section 1. Clinical and healthcare issues	
1A. Related to drug/therapy	
Question	Recommendations
*1. Which drugs are potentially suitable for home administration?*	Recommendation 1.1: ODAH should be limited to parenterally administered drugs (intravenous, intramuscular, or subcutaneous) using electric infusers or elastomeric devices in increasing or decreasing doses. Caregivers should be trained to detect incidents during prolonged infusions, and healthcare personnel should be available if needed. Recommendation 1.2: Drug administration schedule (e.g., daily, weekly, twice per week) and complete duration of treatment (number of cycles) should be considered, but not discriminatory. Recommendation 1.3: Drug administration should take less than 2 hours, including all the steps in the treatment scheme except analytical controls (e.g., previous clinical controls, premedication, drug administration, cleaning and hydration, and aftercare). Longer procedures may be individually considered. Recommendation 1.4: The drug safety profile should be predictable and manageable, and the dispensed drug must not frequently cause pain or infusion-related adverse reactions. Recommendation 1.5: Clinical and analytical controls should be easily performed before administration at the patient’s home, and the results should be available for treatment validation (Pharmacy Service). Recommendation 1.6: Drug quality has to be maintained during production, transport, and at-home administration.

1B. Related to patients	
Question	Recommendations
*2. What profile of patients would benefit from oncology treatment administered at home?*	Recommendation 2.1: It is recommended to initiate the first infusion in the hospital to monitor the adverse reactions such as allergies or anaphylaxis. Recommendation 2.2: Standardized patient selection criteria should be established to ensure safe home-administered oncologic therapies. However, individual characteristics of each patient should also be considered. Recommendation 2.3: A multidisciplinary committee should be responsible for regularly reviewing and revising inclusion and exclusion criteria. An internal procedure should also be established for the rapid assessment and periodic revisions.

1C. Related to healthcare settings	
Question	Recommendations
*3. How ODAH would compromise the efficacy, safety, and quality of life of systemic treatments?*	Recommendation 3.1: Complying with inclusion criteria is crucial for the safe and effective administration of oncology treatment at home. Recommendation 3.2: Standardized protocols and digital platforms utilized in day hospital units can mitigate risks and promote safety for patients and HCPs during validation, preparation, dispensing, and administration. Recommendation 3.3: Drugs that may jeopardize patient safety, quality of life, or treatment efficacy must not be permitted.
*4. What kind of education, accreditation or training should receive the HCPs involved?*	Recommendation 4.1: HCPs involved in ODAH programs require knowledge and experience in drug administration and patient care, along with the ability to coordinate and communicate with various levels of care, both within hospital and territory. Recommendation 4.2: Nurses responsible for ODAH require training in early detection and management of adverse events, extravasations, spills, antineoplastic treatment administration, and home hospitalization. Also, they should have a minimum experience and capacitation according to the particular needs. Recommendation 4.3: The staff involved should receive adequate training to identify socio-family problems and environments that may not be suitable for administering treatment.
*5. What kind of controls should be performed?*	Recommendation 5.1: A consensus manual is essential and should include pre-, during, and post-treatment instructions, signs/symptoms to watch for, transport, handling, disposal of medication and materials, and procedures for responding to adverse reactions, extravasations, spills, or incidents with the central/peripheral venous catheter. Recommendation 5.2: The HaH Service is the responsible of performing the controls during the ODAH procedure (before, during and after administration). These controls should be adequate to patients, their environment, and the administered drug. Recommendation 5.3: The ODAH schedule should be verified by at least two HCPs, especially the infusion rate of the pumps.
*6. What would be the minimum instruments, apparatus or equipment required?*	Recommendation 6.1: It is essential to provide a reference manual detailing procedure. Recommendation 6.2: Ensure proper disposal of essential and additional materials, as well as biological waste by providing containers.
*7. Which aspects should be considered for drug preparation?*	Recommendation 7.1: The chemotherapy should be prepared in the Pharmacy Service following hospital center safety procedures, labelled for home administration. Recommendation 7.2: The risk of exposure to HCPs administering the treatment, patients or caregivers should be minimal. Therefore, treatments must be prepared and conditioned in sterile class II biosafety cabinet; intravenous drugs must be prepared in closed transfer systems and, if possible, will be dispensed with the closed system infusion set purged with the compatible serum; for subcutaneous or intramuscular drugs the syringes will be loaded with safety anti-reflux caps. Recommendation 7.3: Once at patient home, the material should be prepared on an absorbent towel to prevent any type of spill.

### 3.2 Section 2: logistical and administrative issues

**Question 8. To consider the ODAH, what are the requirements that must be fulfilled by the oncology department/hospital?**

**Figure 1:**
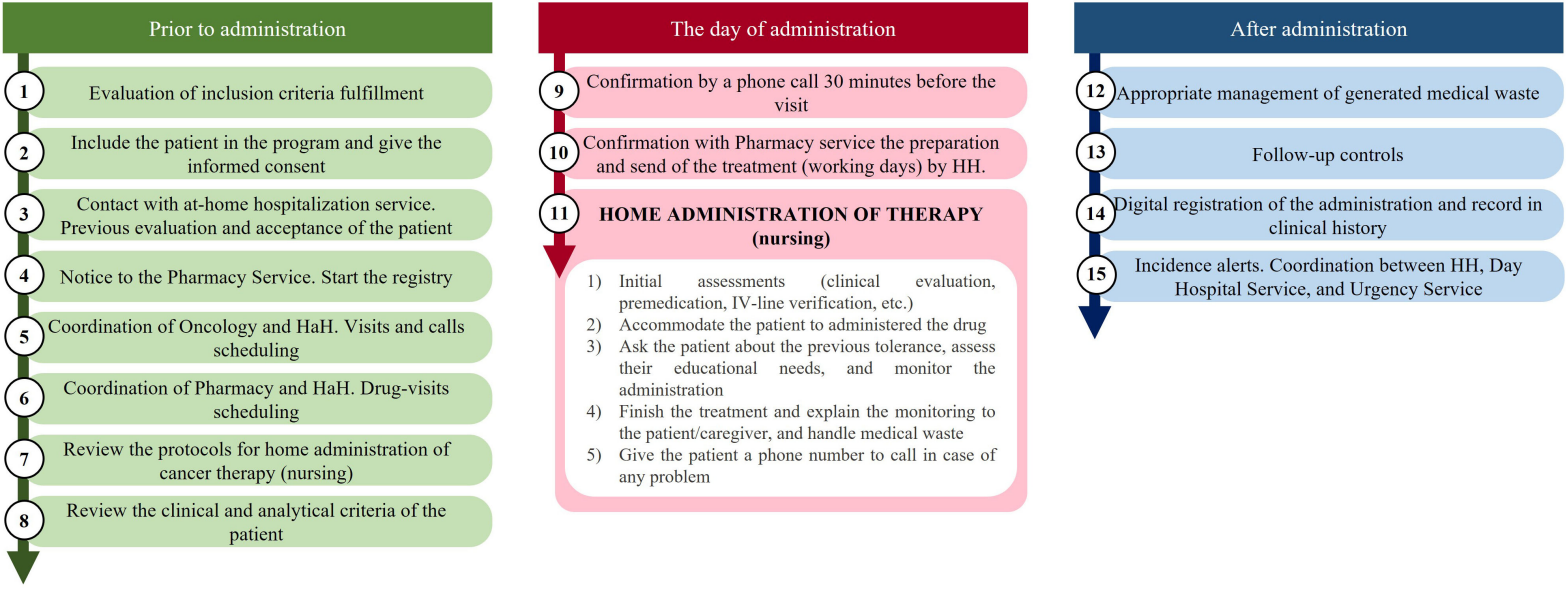
Diagram of the recommended logistic rout for home administration oncologic therapies.

Simplifying the patient’s journey and ensuring the availability of necessary resources and adherence to schedules and processes is crucial. This can be achieved by developing agreed protocols and plans that outline all the steps, stakeholders, and professionals involved. Therefore, it is essential to collaborate and coordinate among nursing, pharmacy, oncology and HaH Services, and all the other involved parties for the program’s success (16, 33, 34). **Figure 1** depicts a recommended circuit model for ODAH.

**Question 9. When should be considered ODAH and for what duration?**

The inclusion of a cancer patient into ODAH will be based on compliance with the defined inclusion criteria. Once the oncologist has approved the treatment and the patient is considered a potential candidate, the Oncology Service is able to coordinate with the HaH Service to determine the patient’s eligibility.

**Question 10. How should the telephone for medical support be implemented?**

A dedicated telephone line for cancer patients has been shown to be effective in reducing unnecessary emergency evaluations and hospital admissions, leading to lower healthcare costs and improved patient quality of life. Oncologists and specialized nurses can detect potential adverse events early, reducing the need for hospital visits, while telephone support can enhance patient-centred care (33, 35).

**Question 11. How could be defined the hospital logistic route?**

Typically, oncology patients follow a pathway for treatment that includes: 1) access/referral to Oncology Service; 2) assessment and decision to treat; 3) patient consent for treatment. In home-based chemotherapy programs, eligibility for home-administered therapy is determined after the treatment decision has been made (16).

#### 3.2.1 Recommendations of the experts panel for the section 2.

**Table 6** contains the agreed recommendations regarding section 2.

**Table 6:** Expert recommendations for logistical and administrative issues.

Section 2. Logistical and administrative issues	
Question	Recommendations
*8. To consider the ODAH, what are the requirements that must be fulfilled by the oncology department/hospital?*	Recommendation 8.1: ODAH programs require the presence of an interdisciplinary HaH Service in the healthcare centre formed by experienced HCPs that comply with the healthcare model of each region. At least one of these professionals must have onco-hematologic training. Recommendation 8.2: The Pharmacy Service should have an oncology pharmacist with expertise in home healthcare protocols, a standardized method to manage medical waste, and an electronic health record system or pharmacotherapeutic history. A teleassistance system or application is also recommended. Recommendation 8.3: The healthcare center should have a significant volume of treatment, extensive experience in outpatient administration of antineoplastic drugs, and a well-established home hospitalization program. Recommendation 8.4: The center should meet the minimum requirements for ensuring quality Service, including maintaining schedules and providing a simple and comfortable patient journey. Recommendation 8.5: In the event of unforeseen circumstances requiring a return of the drug, it should be sent back to the Pharmacy Service.
*9. When should be considered the ODAH and for what duration?*	Recommendation 9.1: Patients can be considered for home-administered chemotherapy from treatment initiation until discontinuation, with agreement from the patient and their caregiver. Recommendation 9.2: Acceptance of home-administered chemotherapy does not preclude hospital visits when necessary. Recommendation 9.3: Discontinuation of home-administered chemotherapy may be decided by the patient/legal representative, the oncology Service (due to adverse events, disease progression, etc.), or the HaH Service (due to non-compliance with inclusion criteria or logistical difficulties).
*10. How should the telephone for medical support be implemented?*	Recommendation 10.1: Medical support telephone should be attended by the oncologist, but internal specialist in HaH Service will visit and treat the patient at home. Recommendation 10.2: The communication between Oncology, Pharmacy, and HaH Services must be direct and fluid throughout the home administration treatment. The involved nurses should have the ability to contact the oncologist directly.
*11. How could be defined the hospital logistic route?*	Recommendation 11.1: The HaH, Pharmacy, and Oncology Services should collaborate closely to provide personalized follow-up for ODAH patients. The HaH Service can also coordinate additional procedures, such as treating infectious complications or providing transfusion support. Recommendation 11.2: The HaH Service should maintain a schedule for patients enrolled in the program and coordinate with the Pharmacy and Oncology Services to ensure proper patient monitoring. Recommendation 11.3: Medical waste management protocols should be adapted for home settings to ensure the safety of patients, caregivers, and HCPs. This involves a thermally insulated, airtight, resistant, and well-labelled cytostatic container, collected upon discharge. The Maintenance Service of the hospital could provide the appropriate container. Recommendation 11.4: Safety incidents must be reported immediately to healthcare authorities due to the biological hazard of the drugs administered.

### 3.3 Section 3: economic, social and legal issues

**Question 12. How can the efficiency of ODAH be determined?**

Health interventions must provide information on their socio-economic value, including the economic impact and whether the additional benefit justifies the cost. Healthcare policy and decision-making processes are recognizing the need to limit resources to finance available interventions while incorporating the concept of opportunity cost from a societal perspective. Therefore, healthcare programs should consider incorporating health outcomes and their incremental costs to provide necessary evidence for evaluating new interventions. A similar methodology should be employed to evaluate the efficiency of ODAH.

Firstly, to evaluate the efficiency of ODAH, the perspective of the analysis has to be established. As ODAH has potential benefits for both patients and the Spanish NHS, the analysis should be conducted from a societal perspective, which considers direct healthcare costs such as the cost of medication, premedication, hydration, and used materials, as well as possible adverse reactions. Direct non-healthcare costs, such as patient or family transportation expenses, the cost of time spent on these trips, and the cost of time spent waiting, should also be considered. Finally, indirect costs such as losses in productivity should be factored in as well.

To optimize ODAH efficiency, experts suggest conducting societal perspective evaluations, which can benefit patients and public healthcare administration by reducing hospital load. For oncological therapies, recommendations include implementing efficiency analysis and considering greater patient comfort and satisfaction, avoiding travel, and reduced Services saturation. If only the Spanish NHS perspective is considered, the cost of home administration may appear higher than hospital administration.

To fulfil the quality-of-life assessment, it is essential to measure outcomes related with patient perception (Patient-Reported Outcomes Measures-PROM/Patient-Reported Experience Measures-PREM) through validated questionnaires (e.g., from CatSalut in the case of Catalonia, or from the Patient Care Unit of the reference hospital). Ultimately, the results obtained in HaH can be compared to those obtained in cost-benefit analysis of the Day Care Hospital.

The implementation of an ODAH offers numerous benefits for patients, caregivers, and society as: (i) it eliminates the need for patients and caregivers to travel to the hospital for oncological therapies, thus saving time and effort; (ii) it prevents work productivity losses for the patient’s family or caregivers who would have otherwise accompanied them to the hospital; (iii) it reduces the care burden at the oncological day hospital, freeing up critical hospital resources; (iv) it potentially improves adherence and persistence to treatment since nursing staff regularly visit the patient’s home. Without home administration, there is a potential risk of non-attendance, which could result in medication non-receipt; (v) patients may experience greater satisfaction with treatment and improved humanization of care, as the home environment reduces the feeling of medicalization and increases comfort during administration, potentially leading to an improvement in health-related quality of life.

**Figure 2:**
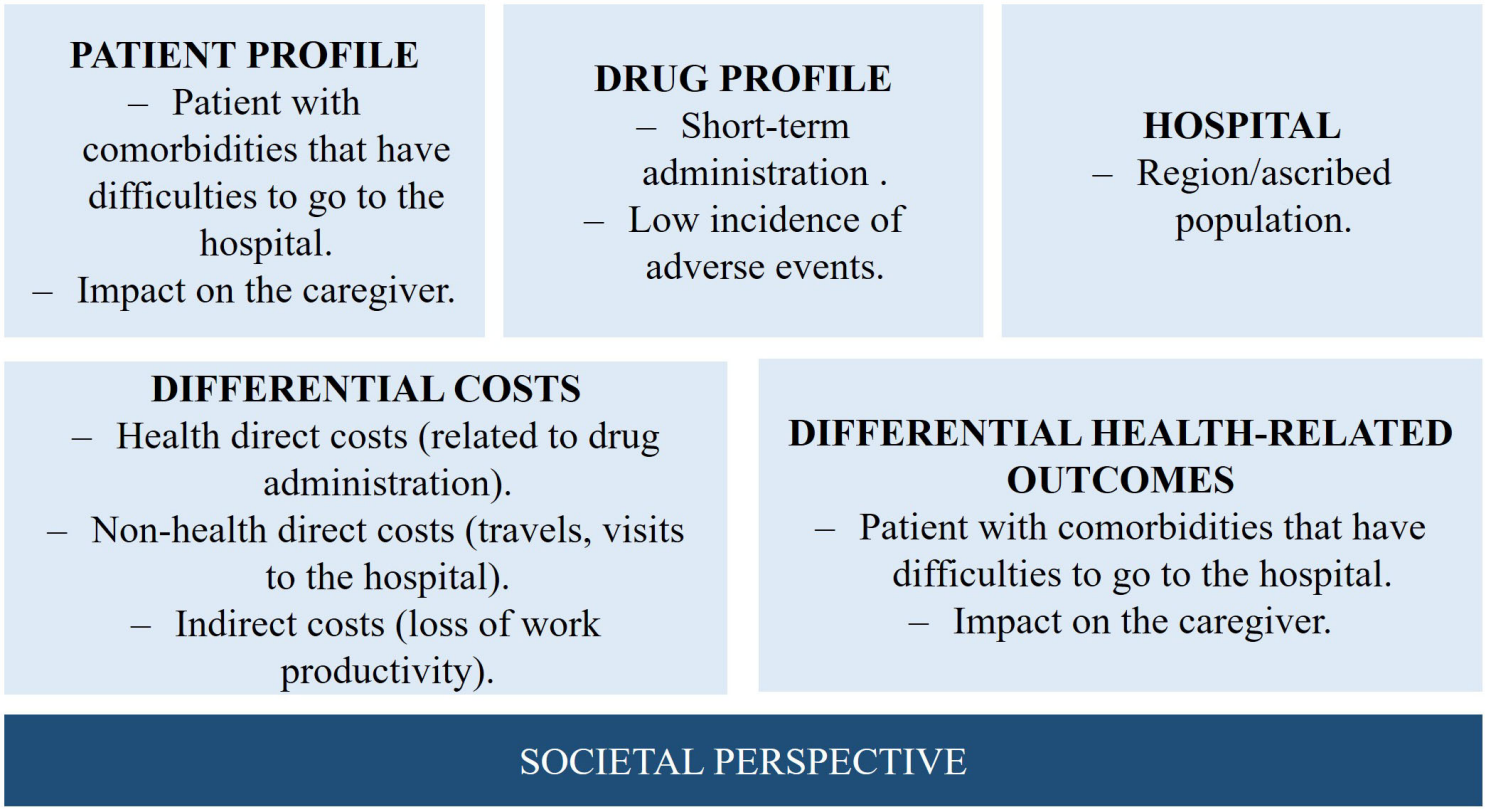
Factors to be considered for the implementation of a pilot study with the aim of assessing the efficiency of the ODAH.

In turn, adequate organizational support, including sufficient budgetary and human resources, is necessary to meet the needs of patients in implementing an ODAH. This includes incorporating qualified technical staff and obtaining appropriate materials to carry out the project effectively. It is also recommended to carry out a pilot program in order to conduct an economic evaluation (**Figure 2**).

Beyond this, other proposals could aid in implementing the ODAH setting, including the possibility of the laboratory covering the differential costs for home administration by sponsoring each patient attended or providing indirect resources, or by removing the VAT associated with drugs for home administration (as done in the United Kingdom) to utilize the potential savings to cover the additional expenses related (18).

In a systematic review by Cool et al. that evaluated the cost efficiency of Oncological Day Hospital at Home (ODAH), nine studies were reviewed (12). Of these, five studies estimate the difference in costs for one home-administered treatment versus hospital-administered from the perspective of the National Health Insurance. These studies reported reductions in costs (ranging from 9% to 53%) that favored home administration (12, 36, 37). Another study evaluated the cost per cycle and resulted in a 3.8% reduction of the costs from a societal perspective (12, 26). Additional systematic review, which included 13 heterogeneous articles, reported that home chemotherapy could result in savings ranging from $1,928 and $2,974 per treatment (38). Recent economic studies also suggest that ODAHs could lead to lower costs (39, 40). Nonetheless, current data do not fully confirm the potential cost reduction derived from home administration, but it is likely that better results of PROMs and PREMs would be obtained (12).

**Question 13: How can the economic impact of ODAH be determined?**

To conform with the guidelines presented in the CatSalut Guide for Economic Evaluations and Budgetary Impact Analyses, it is important to conduct separate assessments of an intervention’s efficiency and its economic impact in order to ensure proper evaluation (41).

As previously mentioned, the societal perspective is the most appropriate since it takes into account the benefits for the patient and their relatives and caregivers. Once the perspective has been defined, costs can be identified, quantified, and evaluated (42).

**Figure 3:**
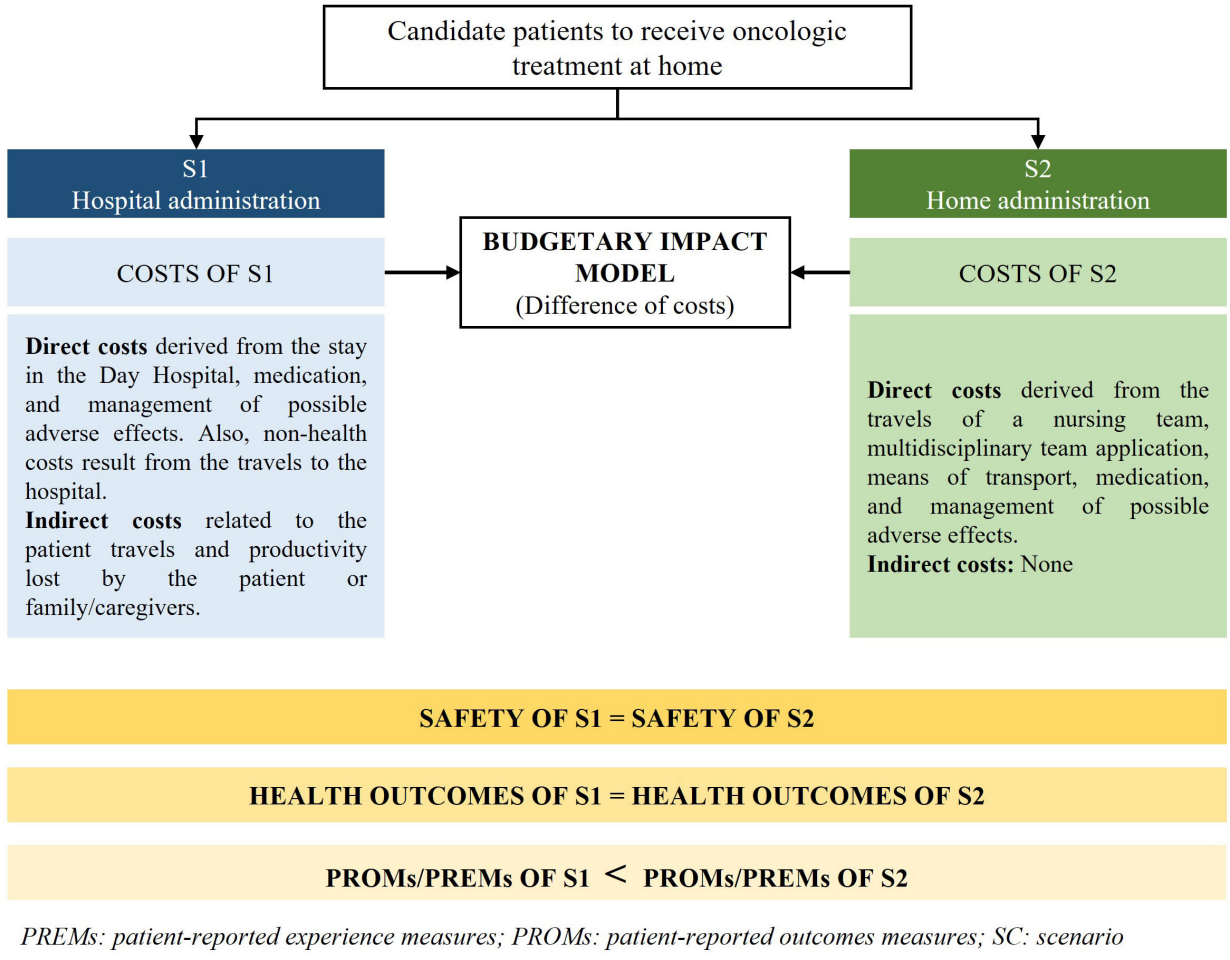
Scheme of the relevant aspects to consider during the economic evaluation of home-administered oncologic therapies.

From a methodological perspective, it is necessary to conduct an economic impact assessment to estimate the difference in costs between ODAH and standard hospital administration (43, 44). To achieve this, two different scenarios should be developed for cancer patients who are suitable for home administration. In the current scenario, named as scenario 1, (S1; hospital administration) all patients received chemotherapy at the hospital, whereas in the potential scenario, named as scenario 2, (S2; home administration) all patients received the treatment at home. The economic impact of ODAH would be the difference between these two scenarios (**Figure 3**). Then, three possible results can be obtained after the estimation: 1) S1 costs higher than S2 costs; 2) S1 costs lower than S2; and 3) the same costs for S1 and S2.

While it would be reasonable to assume that the costs of scenario S1 would be comparable to those of scenario S2, if it is found that the costs of S1 are actually higher than those of S2, this could provide additional support for the adoption of ODAH programs from a healthcare administration perspective.

**Question 14. Are there legal and ethical issues to be solved to implement the program?**

Currently, there is no specific national legislation that regulates the ODAHs. Consequently, HCPs who provide home-administered treatments are legally covered as if they were delivering them at a hospital. Nevertheless, the administration of antineoplastic therapies must be carried out by qualified and experienced professionals to minimize potential risks for the patient and the handler. In case of accidental contamination such as breakage, spillage, or any other incident, the HCPs should be aware of the appropriate measures to manage it. Therefore, the administration of cytostatic agents should be restricted to HCPs who are trained and experienced in the safe handling of chemotherapeutic drugs (45).

#### 3.3.1 Recommendations of the experts panel for the section 3.

**Table 7** summarizes the agreed recommendations for the panel of experts regarding section 3.

**Table 7:** Expert recommendations for economic, social and legal issues.

Section 3. Economic, social and legal issues	
Question	Recommendations
*12. How can the efficiency of ODAH be determined?*	Recommendation 12.1: To evaluate the efficiency of ODAH, it is recommended to conduct an economic analysis from a societal perspective, given the potential benefits it offers to patients and the Spanish NHS. Recommendation 12.2: It is advised to implement a pilot program to perform an economic evaluation of ODAH.
*13. How can the economic impact of ODAH be determined?*	Recommendation 13.1: For economic impact determination, the resulting costs of the ODAH and in the hospital should be compared. Recommendation 13.2: Despite the potential difficulty in measuring PROMs and PREMs, it should be considered in the evaluation. Recommendation 13.3: To adequately capture potential patient benefits associated with home administration, efficiency evaluations should be conducted from a societal perspective. Recommendation 13.4: It is essential to employ validated questionnaires that can effectively gather and assess PROMs and PREMs to generate evidence regarding the health-related outcomes perceived by patients.
*14. Are there legal and ethical issues to be solved to implement the program?*	Recommendation 14.1: ODAH programs should adhere to the legal regulations of the respective country or region to ensure legal coverage for HCPs and procedures. Recommendation 14.2: Standard protocols for collecting and responding to claims should be followed by each center, and patients should be informed through a formal consent before starting treatment. Recommendation 14.3: Efforts should be made to expand the availability of ODAH programs to more patients while ensuring the safety and efficacy of the treatment is not compromised.

## 4 Conclusions

Home-based chemotherapy initiatives have emerged as a viable and safe alternative to traditional hospital treatment for oncology patients. These programs offer several advantages over traditional hospital-based treatment, including increased patient comfort and convenience, improved control of high-risk situations, and reduced costs and hospital overload. While most programs currently operate in urban environments where a high concentration of patients can be found, we believe that this strategy can also be successfully employed in areas with dispersed populations, as long as the travel time does not exceed 30-45 minutes. Furthermore, by providing patient-centered care and reducing the psychological and emotional burden of treatment, ODAH programs have the potential to significantly improve the quality of life of patients with cancer.

To support the development and implementation of these programs, a multidisciplinary group of experts have developed a list of recommendations based on the published literature and the collective expertise of the group aiming to serve as a foundation for the development of future initiatives.

Overall, ODAHs have the potential to revolutionize the way in which oncology patients receive treatment. By providing safe, effective, and patient-centered care, these programs can help to improve the overall experience of cancer treatment for patients and reduce the burden on healthcare systems. Also, an adequate financial investment and the training of specialized teams would be critical. It is therefore important that healthcare providers, policy makers, and other stakeholders work together to support the development and implementation of these programs to improve the quality of life for patients with cancer.

## Data availability statement

The original contributions presented in the study are included in the article/**Supplementary Material.** Further inquiries can be directed to the corresponding author.

## Author contributions

EV: Conceptualization, Methodology, Validation, Writing – review & editing. MA: Conceptualization, Methodology, Validation, Writing – review & editing. MC: Conceptualization, Methodology, Validation, Writing – review & editing. SG: Conceptualization, Methodology, Validation, Writing – review & editing. MEM-M: Conceptualization, Methodology, Validation, Writing – review & editing. MP: Conceptualization, Methodology, Validation, Writing – review & editing. AT: Conceptualization, Methodology, Validation, Writing – review & editing. YS: Conceptualization, Methodology, Validation, Writing – review & editing. MS: Conceptualization, Methodology, Validation, Writing – review & editing.
