# Supplementary material for: National expert consensus on home-administered oncologic therapies in Spain
Source: Front Oncol. 2024 Feb 16;14:1335344. doi: 10.3389/fonc.2024.1335344 (PMC10905380; doi:10.3389/fonc.2024.1335344)
Supplement: Supplementary file 1 [file DataSheet_1.pdf]

Supplementary Material

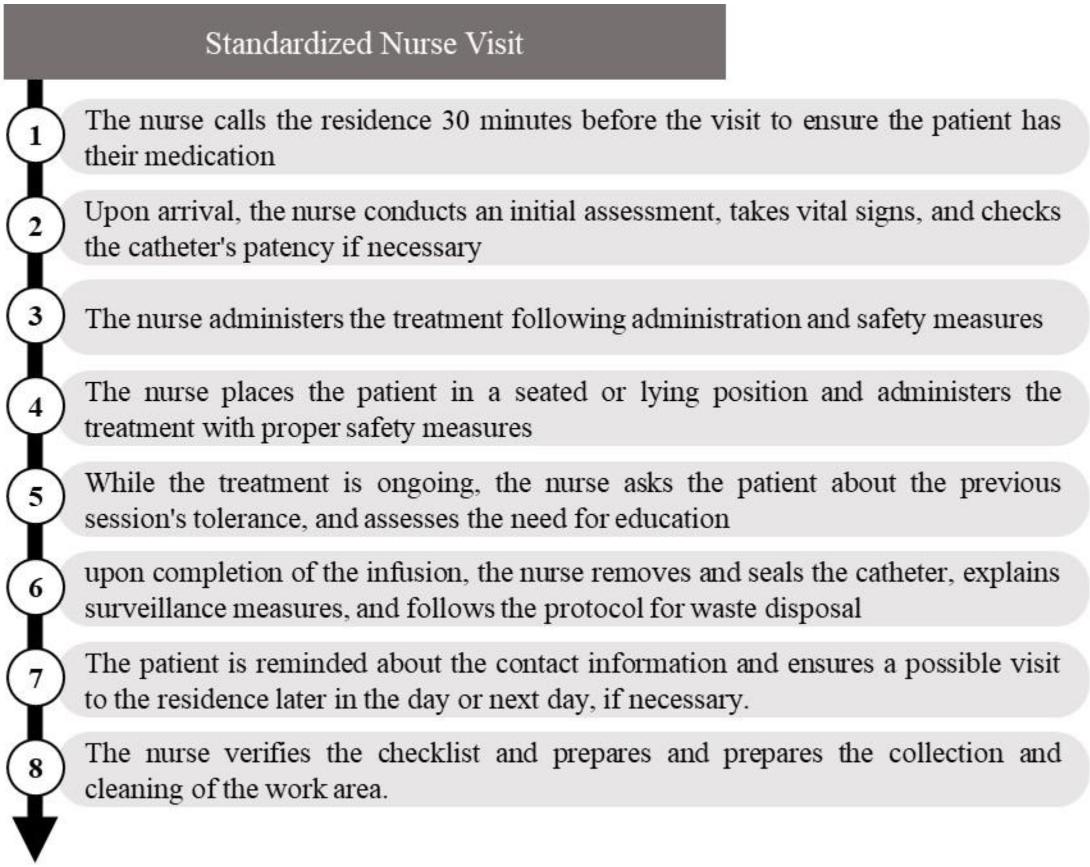

Supplementary Figure S1. Recommended steps for an ODAH nurse visit

Supplementary Table S1. Composition of multidisciplinary groups

CLINICAL AND HEALTHCARE ISSUES	LOGISTICAL AND ADMINISTRATIVE ISSUES	ECONOMIC, SOCIAL, AND LEGAL ISSUES
Estela Moreno (Pharmacist)<br><br>Mª Ángeles Peñuelas (Oncology nurse)<br><br>Eulalia Villegas<br>(Home hospitalization)<br><br>Miguel Ángel Segui (Oncologist)	Sonia González (Pharmacist)<br><br>Ana María Torres<br>(Home hospitalization)<br><br>Eulalia Villegas<br>(Home hospitalization)<br><br>Miguel Ángel Seguí (Oncologist)	Miguel Ángel Casado (Health economist)<br><br>Eulalia Villegas<br>(Home hospitalization)<br><br>Miguel Ángel Seguí (Oncologist)

Supplementary Table S2. Formulated questions for each section

**Section 1. Clinical and healthcare issues**
**1A. Potential drugs**<br><br>– Which drugs are potentially suitable for home administration?<br>**1B. Patient candidates**<br><br>– What profile of patients would benefit from oncology treatment administered at home?<br>**1C. Health care professionals (HCPs) and preparation for home visits**<br><br>– How ODAH would compromise the efficacy, safety, and quality of life of systemic treatments?<br>– What kind of education, accreditation or training should receive the HCPs involved?<br>– What kind of controls should be performed?<br>– What would be the minimum instruments, apparatus or equipment required?<br>– Which aspects should be considered for drug preparation?
**Section 2. Logistical and administrative issues**
– To consider the ODAH, what are the requirements that must be ful-filled by the oncology department/ hospital?<br>– When should be considered the ODAH and for what duration?<br>– How should the telephone for medical support be implemented?<br>– How could be defined the hospital logistic route?
**Section 3. Economic, social, and legal issues**
– How can the efficiency of ODAH be determined?<br>– What are the possible social benefits of oncologic home administration?<br>– Are there legal and ethical issues to be solved to implement the program?

Supplementary Table S3. Main stakeholders involved in home administration of oncologic therapies.

Stakeholder	Role and functions
Medical oncology	– To define the therapeutic strategy.<br>– To propose potential patients for home administration.<br>– Based on therapeutic results, decide if the patient should continue or discontinue the home administration program.
Oncology Pharmacy	– To ensure the maintenance of technical quality of the elaborated drugs from the preparation to the end of the administration.<br>– To prepare and condition drugs for transport.<br>– To ensure the maintenance of drug traceability.<br>– To promote inclusion of new oncologic treatments for home administration (based on e.g., new stability data, route of administration, drug releases in the pharmaceutical market...)
Oncology nursing	– To transport the drug to the home and administrate it to the patient.<br>– Before administration, the nurse is responsible for the interview and clinical assessment to confirm that the appropriate conditions are met (clinical state of the patient, adequate instruments, and home conditions, etc.).<br>– Be responsible for obtaining biological samples necessary for analysis or controls before administration.<br>– Surveillance of the patient immediately after drug administration.<br>– Digital registration of the treatment administration and notification of adverse events that occur at home.<br>– To collect waste after administration and delivery to the hospital.
Home hospitalization team	– To act as a link between the different services involved in home administration programs.<br>– To confirm that the patient meets all inclusion criteria defined for home administration, including a previous home visit.<br>– Be responsible for the logistic organization to optimize the use of the material and human resources available in the centre to attend to as many patients as possible.<br>– Administration of therapy and monitorization after administration following oncologist indications and administration protocols elaborated together with oncology and pharmacy.<br>– Together with nursing, to provide information and education to patients and their relatives/caregivers about home administration and their needs.<br>– To coordinate the initial and continuous training of the professionals involved.<br>– To ensure that periodic quality control processes are completed.<br>– To develop care protocols that will serve as a mandatory guide for the activities included in the program.
Central laboratories of the hospital	– To provide the necessary support for diagnostic and other analytical procedures during home administration.<br>– To ensure the delivery of results on time to avoid delays and to alert about significant alterations which require the patient’s re-evaluation.
General Hospital Services	– To obtain and maintain the material resources necessary for home administration (medical equipment, drug transport devices and waste collection containers, vehicles used for the movement of HCPs, etc).
Clinical documentation Service	– To ensure that all pre- and post-administration records are integrated into the patient's clinical history.

Medical direction of the hospital	– To assess the need for a home administration program for oncology patients and periodically monitor the results to implement some modifications.
Other HCPs (social workers, clinical psychologists, medical educators, etc.)	– To provide support, punctual or regular, as required by the rest of the professionals and Services involved in oncologic home administration.
Patients and caregivers	– To compromise with the objectives of the project and be co-responsible for the follow-up of the results.<br>– To notify about any adverse event that occurs during the procedure.
